# Supplementary material for: Formation Pathways of Lath-Shaped WO3 Nanosheets and Elemental W Nanoparticles from Heating of WO3 Nanocrystals Studied via In Situ TEM
Source: Materials (Basel). 2023 Feb 2;16(3):1291. doi: 10.3390/ma16031291 (PMC9920553; doi:10.3390/ma16031291)
Supplement: Supplementary file 1 [file materials-16-01291-s001.zip › materials-2195179-supplementary.pdf]

Supplementary Materials

Formation Pathways of Lath-Shaped WO_3 Nanosheets and Elemental W Nanoparticles from Heating of WO_3 Nanocrystals Studied via In Situ TEM

Xiaodan Chen [1,2,*] and Marijn A. van Huis [1,2]

1 Soft Condensed Matter, Debye Institute for Nanomaterials Science, Utrecht University, Princetonplein 5, 3584 CC Utrecht, The Netherlands
2 Electron Microscopy Center, Utrecht University, Universiteitsweg 99, 3584 CG Utrecht, The Netherlands
* Correspondence: x.chen1@uu.nl

Supporting Table

Table S1. Structural details including lattice parameters of WO_x phases and of cubic W. The reference code specifies the JCPDS index.

Chemical formula	Reference code	Crystal system	Space group	Lattice parameters					
				a (Å)	b (Å)	c (Å)	α (°)	β (°)	γ (°)
WO_3	00-041-0905	Cubic	$Pm\bar{3}m$	3.71	3.71	3.71	90	90	90
WO_3	00-043-1035	Monoclinic	$P2_1/n$	7.30	7.54	7.69	90	90.91	90
WO_3	01-089-4479	Orthorhombic	Pbcn	7.36	7.57	7.76	90	90	90
WO_3	00-005-0388	Tetragonal	P4/nmm	5.25	5.25	3.91	90	90	90
$WO_{2.72}$	01-073-2177	Monoclinic	P2/m	18.32	3.79	14.04	90	115.2	90
W	00-001-1203	Cubic	Im3m	3.15	3.15	3.15	90	90	90

Supporting Figures

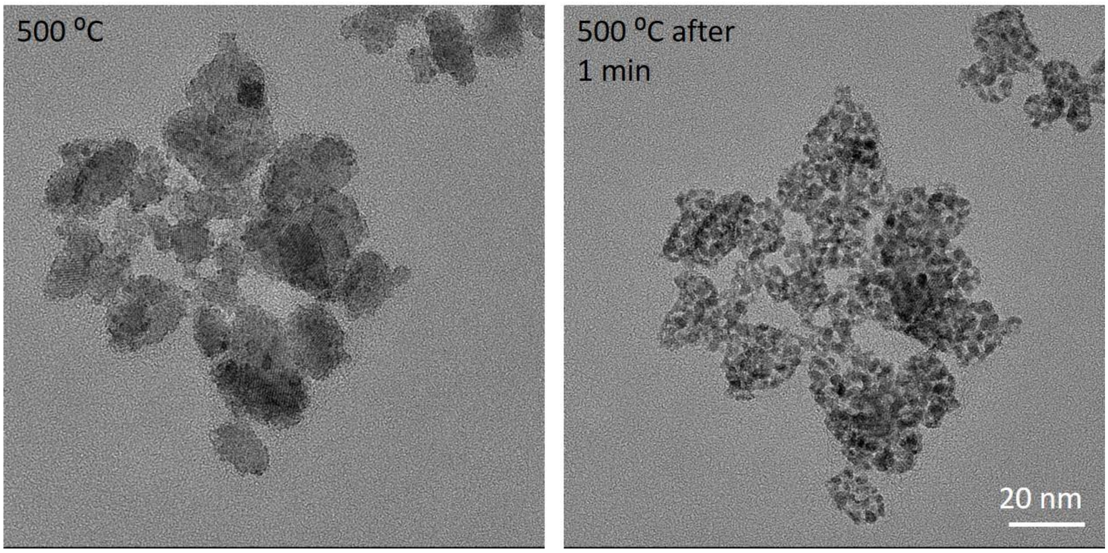

Figure S1. TEM images demonstrating the influence of a high-intensity electron beam during imaging. Bright-field TEM images of WO_3 particles at 500 °C before (left) and after (right) high-intensity illumination by the electron beam.

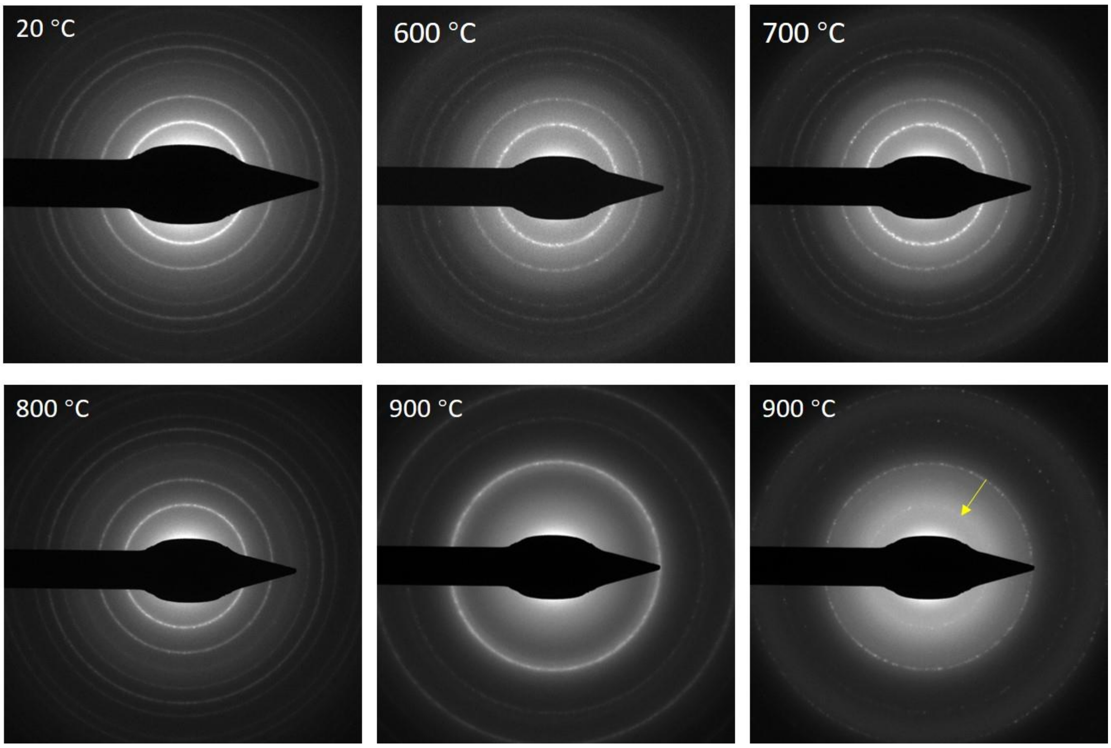

Figure S2. SADPs of WO_x nanocrystals during heating up to 900 °C. Upon heating to 800 °C, the patterns corresponded to the cubic WO_3 crystal structure. The phase transformation to cubic W took place at 900 °C. At some areas where lath-shaped particles formed, the peaks corresponding to WO_3 (marked with yellow arrow in right-bottom image) remained at 900 °C. .

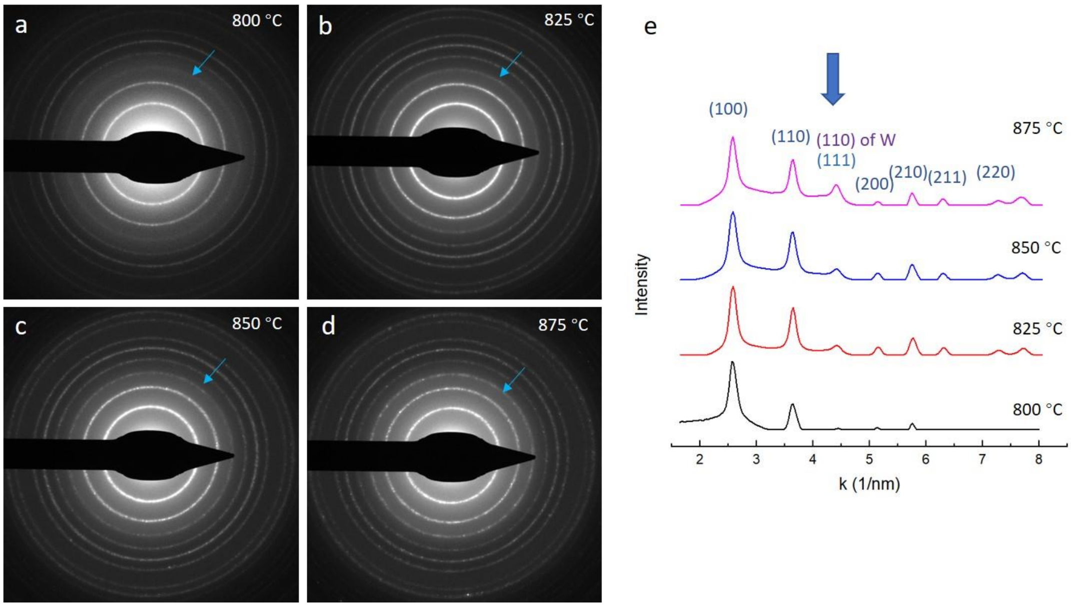

Figure S3. (a)-(d) SADPs of the specimen heated from 800 °C to 875 °C. The blue arrow marks the ring corresponding to (111) reflection planes of WO_3 and the (110)-reflection of W, of which the intensity increased gradually. (e) integrated graphs of DPs in (a) to (d).

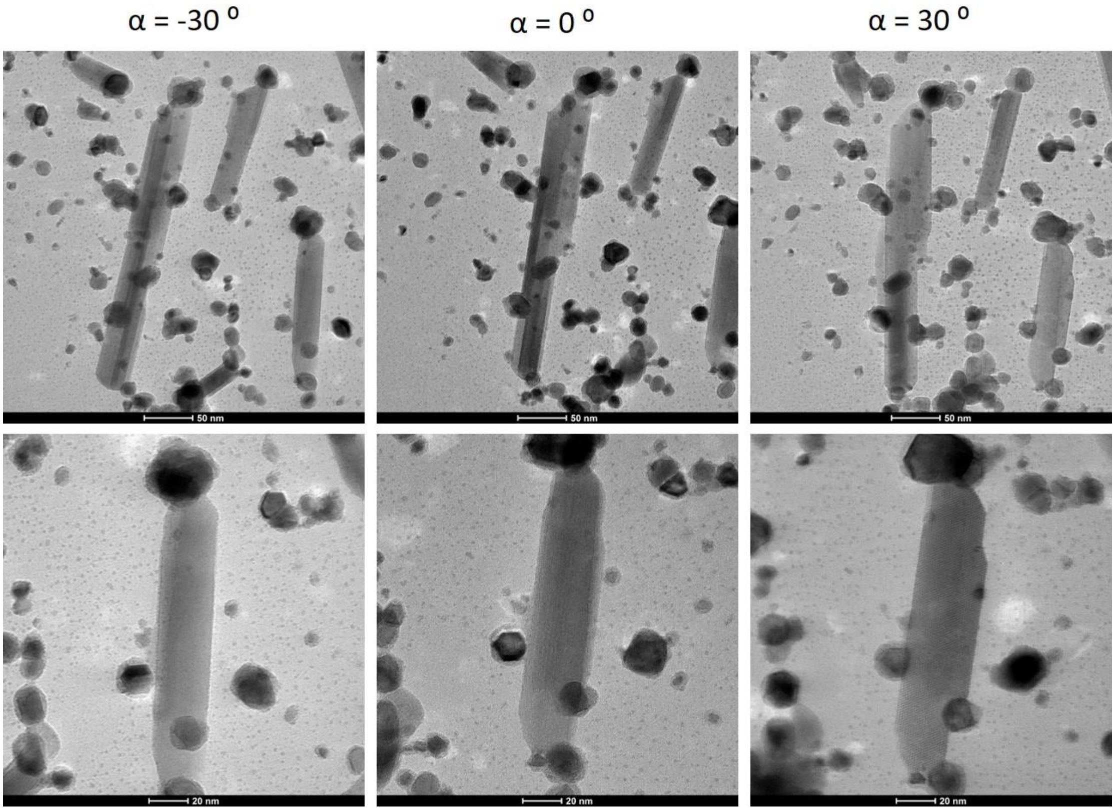

Figure S4. Bright-field TEM images of lath-shaped particles tilted to angles of ±30°. The widths of the particles change with tilting, which indicates that particles are curled up, but not shaped as perfect cylinders.

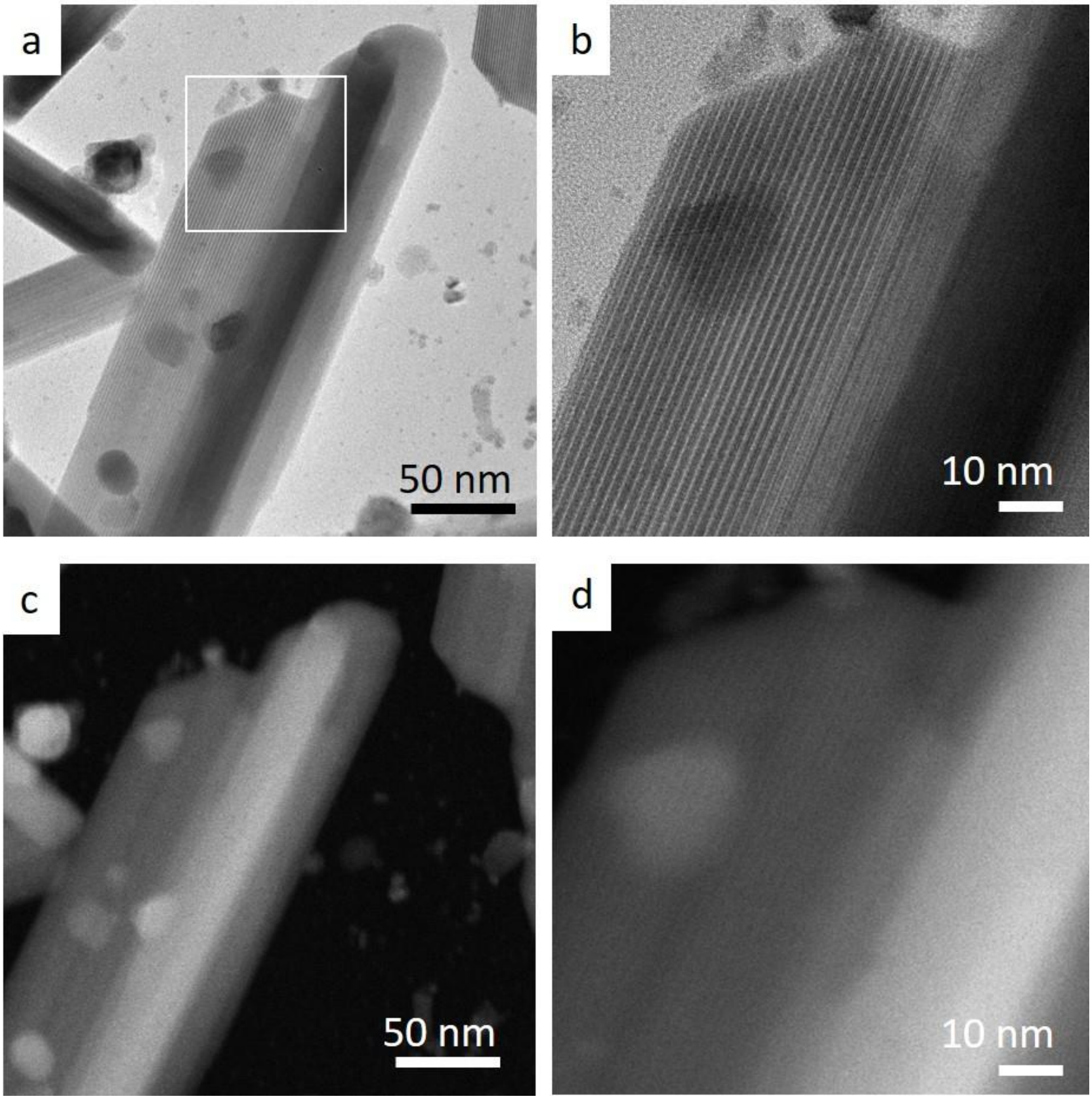

Figure S5. STEM images of lath-shaped particles. (a) Bright-filed TEM image; (b) TEM image of the area marked with a white square in (a). Moiré patterns showing the multiple layers; (c) STEM image with the same magnification of (a); (d) STEM image with the same magnification as in (b).

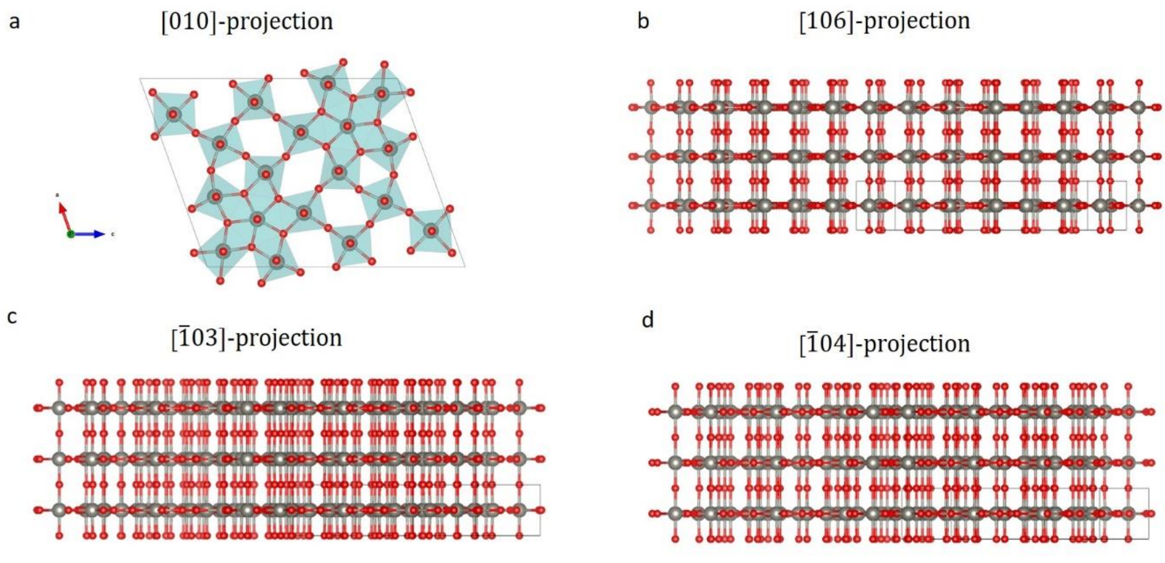

Figure S6. Schematic structure of $WO_{2.72}$ shown in different projections. (a) [010]-projection shown with polyhedral; (b) [106]-projection; (c) [$\bar{1}$03]-projection; (d) [$\bar{1}$04]-projection.
